# Artificial Intelligence-Based Algorithm for Stent Coverage Assessments

**DOI:** 10.3390/jpm15040151

**Published:** 2025-04-11

**Authors:** Joanna Fluder-Wlodarczyk, Mikhail Darakhovich, Zofia Schneider, Magda Roleder-Dylewska, Magdalena Dobrolińska, Tomasz Pawłowski, Wojciech Wojakowski, Pawel Gasior, Elżbieta Pociask

**Affiliations:** 1 Division of Cardiology and Structural Heart Diseases, Medical University of Silesia in Katowice, 40-635 Katowice, Poland; magda.roleder-dylewska@sum.edu.pl (M.R.-D.);; 2 Department of Biocybernetics and Biomedical Engineering, AGH University of Kraków, 30-059 Kraków, Poland; mikhail.darakhovich@gmail.com (M.D.);; 3 Faculty of Geology, Geophysics and Environmental Protection, AGH University of Kraków, 30-059 Krakow, Poland

**Keywords:** personalized medicine, artificial intelligence, percutaneous coronary intervention

## Abstract

**Background:** Neointimal formation after stent implantation is an important prognostic factor since delayed healing may lead to stent thrombosis. In vivo, optical coherence tomography (OCT) can most precisely assess stent strut coverage. Since analyzing neointimal coverage is time-consuming, artificial intelligence (AI) may offer valuable assistance. This study presents the preliminary results of the AI-based tool’s performance in detecting and categorizing struts as covered and uncovered. **Methods:** The algorithm was developed using the YOLO11 (You Only Look Once) neural networks. The first step was preprocessing, then data augmentation techniques were implemented, and the model was trained. Twenty OCT pullbacks were used during model training, and two OCT pullbacks were used in the final validation. **Results:** The presented tool’s performance was validated against two analysts’ consensus. Both analysts showed moderate intraobserver agreement (κ = 0.57 for analyst 1 and κ = 0.533 for analyst 2) and fair agreement with each other (κ = 0.389). The algorithm’s detection of struts was satisfactory (a 92% positive predictive value (PPV) and a 90% true positive rate (TPR)) and was more accurate in recognizing covered struts (an 81% PPV and an 85% TPR) than uncovered struts (a 73% PPV and a 60% TPR). The agreement was κ = 0.444. **Conclusions**: The initial results demonstrated a good detection of struts with a more challenging uncovered strut classification. Further clinical studies with a larger sample size are needed to improve the proposed tool.

## 1. Introduction

The standard treatment for patients with symptomatic chronic coronary syndrome (CCS) and acute coronary syndrome (ACS) is percutaneous coronary intervention (PCI) with drug-eluting stent (DES) implantation. Neointimal formation following stent deployment is an important prognostic factor for these procedures. Excessive endothelial growth can lead to restenosis, requiring subsequent interventions [1], while delayed healing may lead to stent thrombosis (ST) [2,3], which is associated with significant morbidity and mortality [4]. The imaging modality that is destined for an in vivo assessment of stent struts’ neointimal coverage is optical coherence tomography (OCT). OCT provides high-resolution cross-sectional images of both native coronary arteries and implanted stents, enabling visualization of microscopic structures [5,6,7]. However, analysis of neointimal coverage is time-consuming and requires significant interpretation skills preceded by extensive education and training, making it useless in daily clinical practice and challenging and expensive in experimental settings. Currently, most OCT analyses are conducted by CoreLabs. These limitations have raised the urgent need for automated strut-level classification algorithms [8].

Artificial intelligence (AI) has made remarkable progress recently, finding applications also in healthcare. Particularly interesting is the use of AI for repetitive and tedious tasks. Deep learning, one of the AI domains, uses artificial neural networks (ANN) to imitate the human brain in processing data and creating patterns. Different types of ANNs are available depending on the specific problem. Convolutional neural networks (CNNs), such as YOLO (You Only Look Once) networks, are especially effective in finding patterns in images and recognizing objects, classes, and categories [9].

Previously, we presented an algorithm developed by engineers from the AGH University of Kraków for the automated quantitative analysis of vessel lumen and stent struts at the early stages of vessel healing in intravascular OCT imaging. The algorithm demonstrated excellent agreement with a manual estimation of the lumen and stent area. However, the quantification of strut coverage was more challenging [10]. We have aimed to improve the algorithm using AI to enhance its performance in classifying struts. This study presents the preliminary results of the algorithm’s performance in detecting and categorizing struts as covered and uncovered.

## 2. Materials and Methods

### 2.1. Study Description

We selected 22 suitable OCT examinations. We included only OCTs with visible stents in the early stages of healing. Of these, 19 pullbacks were from patients who underwent an OCT imaging follow-up one month (on average 32 ± 3 days) after an OCT-guided stent deployment. The remaining three OCT pullbacks were performed for clinical indications on an average of 97 days (75, 149, and 66 days) after PCI. Poor-quality pullbacks were excluded, especially those with a significant amount of residual blood. All procedures were performed in the Division of Cardiology and Structural Heart Diseases, Medical University of Silesia in Katowice. All patients provided written informed consent for the OCT examination. Due to the study’s retrospective nature and lack of interference in the diagnostic and therapeutic decision-making processes, no permission was required from the Institutional Review Board and Bioethics Committee, but the Local Bioethical Committee approved OCT imaging one month following DES implantation. The data were anonymized prior to analysis. OCT was performed using the ILUMIEN OPTIS system (Abbott Vascular, Santa Clara, CA, USA). The examination included the entire stented segment as well as 5 mm proximally and distally to the stent. OCT registration was preceded by a contrast injection, which triggered an automatic pullback. Patients received unfractionated heparin prior to the procedure to obtain an activated clotting time (ACT) of >250 s. The analyses were performed at intervals of 0.2 mm. Patients’ characteristics are summarized in Table 1.

The dataset was divided into three subsets: the training set (17 pullbacks), the validation set (3 pullbacks), and the testing set (2 pullbacks—one 75 days and the second 28 days after stent implantation). Each pullback represents a sequence of OCT images along a coronary artery segment. Dividing the data in this manner ensures that the model is trained and validated on distinct anatomical regions, reducing the risk of overfitting and providing a robust performance evaluation. The final validation and performance metrics were assessed on the testing dataset, which was not used during training or validation.

The training set consisted of 2312 frames. Only 10% of the frames had no visible struts. The validation (1017 frames) and testing (644 frames) sets included whole pullbacks. During training, a validation set was used to monitor the model’s performance and adjust the hyperparameters accordingly. We employed the Ultralytics YOLO framework, which relies on a comprehensive fitness metric derived from the mean Average Precision (mAP). This metric, known for its robustness in evaluating object detection performance across varying confidence thresholds, was monitored after each epoch.

Validation metrics guided decisions on learning rate adjustments and potential early stopping to prevent overfitting. The last two pullbacks were used in the testing set. These examinations were reserved exclusively for the final evaluation to assess the model’s generalization to unseen data. No frames were excluded from the testing, demonstrating the varying degrees of image quality encountered in daily clinical practice. In each image, all struts were categorized as either covered or uncovered (defined as a strut not covered by tissue at any side or covered only at one side). The annotation process was performed using the Computer Vision Annotation Tool (CVAT.ai) [11]. The training set was divided among four analysts (three cardiologists experienced in OCT analysis and one CoreLab analyst). The validation set was assessed by two analysts (one cardiologist and a CoreLab analyst) and reviewed by them to obtain their consensus. The testing set was initially analyzed by the AI model. Next, two analysts were asked to review and edit the data offered by the tool. The assessments were performed twice, with at least a week’s break between analyses. Then, the intraobserver agreement was calculated, and the analysts could again categorize false negative and false positive struts, obtaining a ground truth (GT) for each analyst. The GT refers to the definitive classification of each stent strut as either “covered” or “uncovered”. The next step was a comparison between analysts, achieving interobserver agreement. Both analysts reviewed the false negative and false positive struts, establishing the final label for differing struts. This consensus of two analysts was used as a GT for the algorithm evaluation.

### 2.2. Data Preprocessing, Model Architecture, and Training

In this study, we explored the application of neural networks in medical imaging to detect metallic struts in coronary arteries using OCT images. We employed the YOLO (You Only Look Once) family of object detection algorithms, specifically testing versions YOLO8 through YOLO11. Among these, YOLO11 demonstrated superior performance in accurately identifying metallic struts.

The first step was preprocessing. The OCT images were originally acquired and processed in Cartesian coordinates with 704 × 704 pixels dimensions. Pixel intensity values were normalized to the [0, 1] range to facilitate faster convergence during training. Then, data augmentation techniques were implemented to enhance model generalization. These included geometric transformations (rotation, scaling, horizontal and vertical flip) and color transformations.

Next, we utilized the pre-trained YOLO11x model as the foundation for our object detection task. The YOLO11 architecture was developed and provided by Ultralytics, and it is distributed under the GNU Affero General Public License v3.0 (AGPL-3.0) [12]. Training was conducted on a single NVIDIA A100 GPU with 40GB of memory. This model was initially trained on the COCO2017 dataset, which contains over 200,000 labeled images across 80 object categories. Training on COCO2017 provides a robust starting point due to its diverse feature representations and extensive object instances. Leveraging transfer learning, we fine-tuned the YOLO11x model to adapt it to our specific application. The model was customized to detect two classes of metallic struts: covered and uncovered. The final layers of the network were modified to output predictions for the two targeted classes instead of the original 80 classes from COCO2017. The architecture incorporates advanced convolutional layers, residual connections, and attention mechanisms to enhance feature representation and localization precision.

### 2.3. Statistic Analysis

In object detection, evaluation metrics such as the PPV (Positive Predictive Value − Precision), TPR (True Positive Rate − Recall), and F1-score are widely used to assess performance:

PPV = TP/(TP + FP)

TPR = TP/(TP + FN)

F1-Score = 2 × (PPV × TPR)/(PPV + TPR)

where TP (True Positive) represents the number of correctly identified stent struts, FP (False Positive) refers to the non-stent struts incorrectly classified as stent struts, and FN (False Negative) corresponds to the actual stent struts that the model failed to detect.

PPV reflects the proportion of correctly detected stent struts among all predicted stent struts, while TPR quantifies the fraction of actual stent struts that were successfully identified. The F1-score serves as a harmonic mean of the PPV and TPR, offering a comprehensive performance measure. This metric is particularly useful when the classes are imbalanced because it accounts for false positives and false negatives, providing a balanced measure of the algorithm’s accuracy.

The kappa statistic was used to evaluate inter-rater reliability. Specifically, Cohen’s kappa was applied to assess the level of agreement between two analysts. This statistic accounts for agreement occurring beyond chance, providing a more accurate measure of reliability in categorical assessments.

## 3. Results

**Figure 1:**
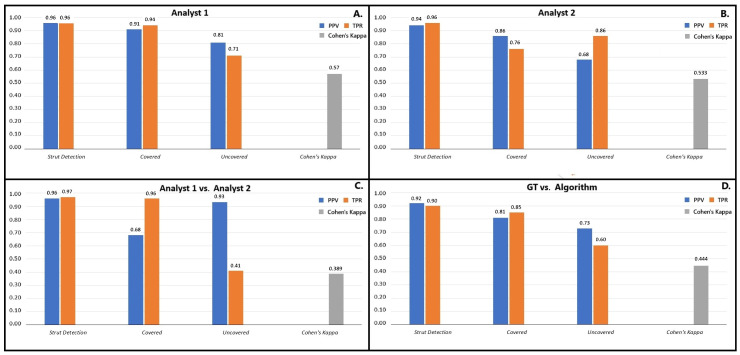
Summary of intraobserver variability for analyst 1 (**A**), analyst 2 (**B**), interobserver variability (**C**), and algorithm performance versus ground truth (GT)—consensus for analysts 1 and 2 (**D**).

A comparison of analyst 1’s assessments demonstrates high consistency in detecting struts (96% PPV and 96% TPR) and recognizing covered struts (91% PPV and 94% TPR) but was lower for uncovered struts (81% PPV and 71% TPR). The intraobserver agreement for analyst 1 was κ= 0.57 (a 95% confidence interval from 0.538 to 0.602). Analyst 2 was slightly less repeatable (86% and 68% PPV and 76% and 86% TPR for detecting covered and uncovered struts, respectively). The overall detection of struts was excellent (94% PPV and 96% TPR). The intraobserver reliability for analyst 2 was κ= 0.533 (a 95% confidence interval from 0.509 to 0.558). The next step was to compare the results of analyst 1 and analyst 2. The interobserver agreement was κ= 0.389 (a 95% confidence interval from 0.364 to 0.414). Compared to analyst 1, analyst 2 demonstrated 68% PPV and 96% TPR in detecting covered struts and 93% PPV and 41% TPR for uncovered struts. A high consistency between both analysts was observed in total strut detection (96% PPV and 97% TPR). These findings are summarized in Figure 1. Analyst 2 agreed better with the GT (κ = 0.693, with a 95% confidence interval from 0.670 to 0.715, 95% and 77% PPV, and 83% and 91% TPR for the detection of covered and uncovered struts, respectively) than analyst 1 (κ = 0.558, with a 95% confidence interval from 0.531 to 0.585, 80% and 98% PPV, and 98% and 51% TPR for covered and uncovered struts).

**Figure 2:**
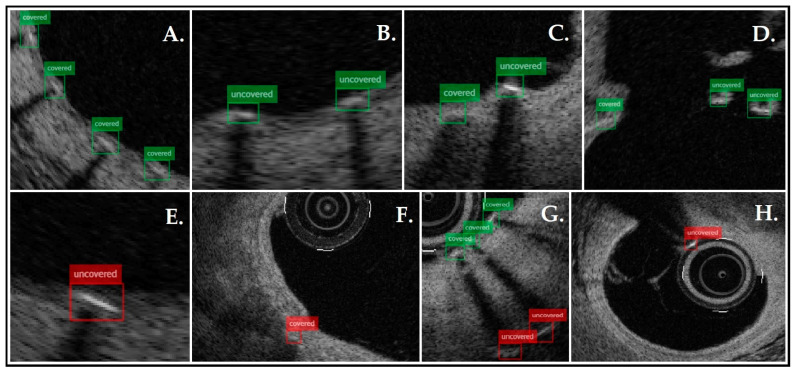
Example frames analyzed by the presented tool: (**A**–**D**)—shows the correct detection and classification of struts; (**E**)—a thinly covered strut was incorrectly classified as uncovered; (**F**)—calcification was confused with a covered strut; (**G**)—ghost strut artifacts (multiplied struts in the shadow area) were confused with an uncovered strut; and (**H**)—a catheter was incorrectly identified as an uncovered strut.

Figure 2 shows the sample frames analyzed by the presented tool, presenting correctly and incorrectly labeled struts. Analyses were rapid and efficient; the average time needed for one pullback evaluation was 30 s. The performance of the presented tool was validated against GT. The algorithm identified 3439 struts, of which 2440 were classified as covered and 999 uncovered, while GT detected 3539 struts, 2324 covered and 1215 uncovered. Detection of the struts was very good (92% PPV, 90% TPR, and 91% F1-score). The algorithm was more accurate in recognizing covered struts (81% PPV, 85% TPR, and 83% F1-score) than uncovered struts (73% PPV, 60% TPR, and 66% F1-score). The agreement was κ = 0.444 (a 95% confidence interval from 0.420 to 0.468). Table 2 and Figure 1 present the described data.

## 4. Discussion

**Figure 3:**
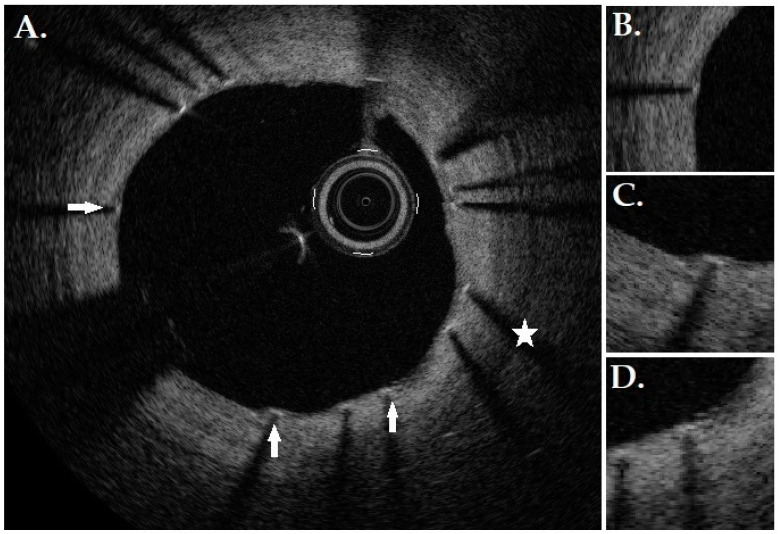
(**A**) OCT frame from 1-month follow-up examination. Most visible struts are covered (an example is marked with an asterisk). However, it is questionable whether several struts are thinly covered or uncovered (arrows). (**B**–**D**) The zoomed view of questionable struts.

This study aimed to present the preliminary results of the AI-based tool’s performance. The detection of struts was very good (92% PPV, 90% TPR, and 91% F1-score). The classification of covered struts was also satisfactory (81% PPV, 85% TPR, and 83% F1-score). However, the recognition of uncovered struts proved to be more challenging (73% PPV, 60% TPR, and 66% F1-score). Three main reasons are responsible for this outcome. Firstly, more data are required to enhance the classification of uncovered struts. We are collecting new OCT images since we have already used all suitable examinations. Secondly, classes (covered or uncovered) were unbalanced in the OCT pullbacks used in this study. Most of the struts were covered, which decreased the opportunity for AI to learn to recognize the uncovered struts properly. Lastly, most OCT examinations were from one-month follow-ups, which resulted in many thinly or partly covered struts. Sometimes, the difference between the covered or uncovered label was very discrete, making correct classification more challenging, as presented in Figure 3.

Naturally, it is difficult to avoid comparing the proposed tool with others published previously. Wang et al. presented a tool that detected malapposed, apposed, and covered struts with sensitivities of 91.0%, 93.0%, and 94.0%, respectively [13]. Ughi et al. reported an algorithm that was characterized by high Pearson’s correlation coefficients (R = 0.96–0.97) between the automated and manual measurements of stent strut apposition and strut coverage measurements [14]. An algorithm presented by Nam et al. used ANNs and was efficient in detecting struts and classifying between covered and uncovered struts (TPR and PPV above 90%) [15]. Fully automated machine learning-based software analysis, shown by Lu et al., provided objective, repeatable, and comprehensive stent analyses. The tool was very effective in detecting and classifying struts (sensitivity/specificity of 94%/90% in detecting uncovered struts [16], and in another study with more challenging cases, this was 82%/99% [17]). Comparing the currently presented method with the algorithm previously published by us, we see similar effectiveness in strut detection (the previous algorithm achieved a PPV of 89.7% and a TPR of 91.4%, while the new method shows a PPV of 92% and a TPR of 90%) and the classification of covered struts (87%PPV and 80% TPR vs. 81% PPV and 85% TPR, reported by the presented method). The detection of uncovered struts remains challenging (77.3% PPV and 99% TPR for the previous algorithm vs. 73% PPV and 60% TPR for the current one) [10]. However, comparing the current tool with our previous algorithm, it should be emphasized that the validation process in this study was more complex and reliable. Moreover, several factors might be responsible for the differences between various algorithms. Firstly, each algorithm was developed using different techniques and has its limitations. Validation was performed on different OCT images, the quality and difficulty of which can vary significantly, impacting the algorithms’ performance. Lastly, analyst errors are possible, as discussed in more detail below. What distinguishes the proposed method is its approach to utilizing the latest YOLO11 algorithm with enhanced architecture and other improvements, i.e., better detection of small objects like stent struts. The tool may facilitate coverage analysis even in its current form, but since the performance is not fully satisfactory, we plan to improve it further. For this, more OCT studies will be needed, preferably from different periods of coronary stent healing. Of course, agreement between analysts in categorizing struts as covered or uncovered also affects the algorithm’s performance.

Analyzing the results from automated tools should also account for the possibility of analyst errors. Some fluctuation will always be present, partly because coverage assessment is not fully standardized yet, and analysts might have slightly different perceptions of the strut coverage status. Considering this, we involved four analysts in the training process in order to expose the tool to some variability. Additionally, validation was based on a two-analyst consensus to minimize further errors resulting from analyst misjudgment. Both analysts showed moderate intraobserver agreement (κ = 0.57 for analyst 1 and κ = 0.533 for analyst 2) and fair agreement with each other (κ = 0.389). The differences represent different levels of expertise. Furthermore, the analysts tended to choose other zoom settings, which might affect the results substantially [18]. Analyst 1 zoomed more, leading to better detection of thinly covered struts, but also sometimes falsely perceived partly covered struts as covered. Several studies have assessed intra- and interobserver variability. Antonsen et al. showed excellent intra- and interobserver agreement (κ = 0.91 and κ = 0.88, respectively) [19], and comparably, Matsumoto et al. (κ = 0.82 and κ = 0.75 for intra- and interobserver variability) [20]. Otherwise, Brugaletta et al. reported wide inter- (κ = 0.07–0.69) and intraobserver (κ = 0.37–0.86) agreement for qualitative strut coverage assessment. It is worth emphasizing that two CoreLab analysts and two interventional cardiologists with wide experience in OCT evaluations were involved in the mentioned study [18]. Lu et al. demonstrated interobserver variability with an 80–95% agreement on covered struts and a 60–80% agreement on uncovered struts [16]. These studies demonstrate that OCT is a reliable tool for coverage analysis. However, some mistakes are inevitable, especially for less experienced analysts. We can assume that some of these errors are due to fatigue. Automatic tools for coverage assessments can address this issue because a comparison of fully manual analysis with software and manual editing showed that software assistance greatly improves interobserver strut classification agreement [17].

Automated algorithms accelerate analysis at the strut level, providing many benefits and creating new possibilities. The most innovative concept is DAPT discontinuation, which is based on arterial healing after stent deployment. This is only feasible for specific patients for whom the benefits of undergoing an invasive procedure outweigh the risks. Data regarding OCT-based DAPT cessation are limited. The PROTECT-OCT study tested a population of cancer patients with a recently implanted stent that requires premature DAPT cessation due to cancer-related procedures. After diagnostic coronarography and OCT, low-risk individuals were determined (the criteria included >90% of struts covered, >90% apposition of struts, expansion, absence of in-stent restenosis, or intraluminal masses during OCT examination). These patients safely discontinued DAPT during the cancer-related procedure. However, the study had a limited number of patients (40 patients), which limits the power of the study [21]. Early DAPT discontinuation based on the percentage of uncovered struts at a three-month OCT follow-up in a relatively low-risk population was evaluated in the DETECT-OCT study. Patients were assigned to either three months of DAPT (less than 6% of uncovered struts) or 12 months of DAPT (more than 6% of uncovered struts). Composite events rarely occurred in both groups [22]. The basic challenge in planning such studies is the absence of a precise value of uncovered struts that are associated with adverse clinical outcomes. One study determined that 5.9% of uncovered struts was the best cut-off value in predicting major adverse events. However, the cut-off value was derived from patients who experienced MACE, and their number was limited (six patients) [23]. Furthermore, the prognostic factor for ST is not only the overall percentage but also the spatial accumulation of uncovered struts [3,24]. Also, it must be emphasized that a covered strut in OCT is not synonymous with optimal endothelialization, but qualitative neointimal characterization may provide additional information. The homogeneous high-intensity pattern overall represents maturing neointimal tissue [25]. The OCT study comparing vascular healing after implantation of durable- or biodegradable-polymer DES showed, at the 3- and 18-month follow-ups, a high percentage of neointimal frames with homogenous high-intensity signal patterns in both platforms. This suggests favorable vascular responses after PCI with biocompatible polymers, regardless of whether they are durable or biodegradable [26]. Finally, delayed arterial healing is just one of the ST determinants. ST is associated with many risk factors, such as age, a history of prior MI, congestive heart failure, low hemoglobin, or diabetes mellitus [27,28]

Another benefit of automatic algorithms is the increased analysis intervals, which are crucial factors that influence the results. Large intervals (0.5–1 mm) are suitable for assessing the lumen and stent area, while smaller intervals are required for strut coverage assessments since larger intervals may lead to higher variability [29]. Automated algorithms enable the analysis of all available frames in a short time. Proper assessment of stent coverage is especially crucial for patients presenting with myocardial infarction, who might experience delayed endothelialization at culprit sites compared with patients with stable angina [30,31].

## 5. Limitations

This study has several limitations. First, the sample size was limited. Adding more OCT cases to the training set may increase the algorithm’s efficiency. Second, the algorithm was not trained and tested for multi-layer stent strut classification, which makes the proposed tool unsuitable for some patients. The proposed tool does not recognize strut aposition. Additionally, the spatial distribution of uncovered struts is not examined by the algorithm, which is also important information in terms of thrombotic complications [3]. Finally, the algorithm was not directly compared with other similar software, and the proposed method has not been validated histologically.

## 6. Conclusions

This paper introduces an AI-based method for quantitative stent strut coverage assessment. The initial results demonstrated a good detection of struts, with more challenging uncovered strut classification. Further clinical studies with a larger sample size are needed to improve the proposed tool. Automatic methods might be a promising alternative that enhances and facilitates OCT analysis.

## Figures and Tables

**Table 1:** Patients and procedural characteristics.

	All Pullbacks (n = 22)	The Testing Set (n = 2)
Age (average)	68	63
Male	14	1
Indication for PCI		
ACS	3	1
UA	10	0
CCS	9	1
Risk factors		
Hypertension	18	2
Diabetes mellitus	4	0
Dyslipidemia	6	0
Smoking	5	2
Coronary artery		
LAD	8	2
Cx	8	0
IM	1	0
RCA	5	0
Stent type (strut thickness)		
Alex Plus (71 µm)	9	0
Resolute Onyx (81 μm)	8	0
Supraflex Cruz (60 μm)	2	1
Resolute Integrity (90 μm)	1	0
Orsiro (60 μm)	2	1

ACS—acute coronary syndrome, UA—unstable angina, CCS—chronic coronary syndrome, PCI- percutaneous coronary intervention, LAD—left anterior descending artery, CX—circumflex artery, IM—intermediate artery, RCA—right coronary artery.

**Table 2:** Comparison of GT and AI model’s performance.

	GT	Algorithm	GT vs. Algorithm	
			PPV (%)	TPR (%)
Total strut	3539	3439	92	90
Covered	2324	2440	81	85
Uncovered	1215	999	73	60

GT—ground truth; PPV—positive predictive value; TPR—true positive rate.

## Data Availability

The raw data supporting the conclusions of this article will be made available by the authors upon request.
